# Research Progress on the Use of Metformin in Leukemia Treatment

**DOI:** 10.1007/s11864-024-01179-3

**Published:** 2024-01-30

**Authors:** Qian Wang, Xudong Wei

**Affiliations:** Department of Hematology, The Affiliated Cancer Hospital of Zhengzhou University & Henan Cancer Hospital, Zhengzhou, 450008 China

**Keywords:** Leukemia, Metformin, AMPK

## Abstract

Metformin is a first-line drug in the clinical treatment of type 2 diabetes. Its main molecular mechanism involves the activation of adenosine 5′-monophosphate-activated protein kinase (AMPK), which regulates cell energy metabolism. Many clinical studies have shown that metformin can reduce the incidence and mortality of cancer in patients with or without diabetes. In vitro studies also confirmed that metformin can inhibit proliferation, promote apoptosis, and enhance the response of cells to chemical drugs and other anticancer effects on a variety of leukemia cells. In recent years, leukemia has become one of the most common malignant diseases. Although great progress has been made in therapeutic approaches for leukemia, novel drugs and better treatments are still needed to improve the therapeutic efficacy of these treatments. This article reviews the application status and possible mechanism of metformin in the treatment of leukemia to further understand the anticancer mechanism of metformin and expand its clinical application.

## Introduction

Leukemia is a hematopoietic system disease caused by the abnormal proliferation, enhanced self-renewal ability, blocked differentiation, and reduced apoptosis of leukemic cells caused by hematopoietic cell mutations [1]. Leukemia accounts for 2.8% of all new cancer cases and 3.4% of new cancer deaths [2]. At present, leukemia is still treated mainly by combined chemotherapy, but these traditional treatments have serious side effects and can lead to drug resistance. Inter- and intra-tumor heterogeneity (ITH) of leukemia has been indicated as a major obstacle to effective treatment. Although a variety of targeted therapies and immunotherapies can improve the treatment efficacy for leukemia, the prognosis of patients with recurrent leukemia is still poor, revealing the urgent need for further leukemia study and novel treatment opportunities.

Metformin is a lipophilic biguanide compound that can inhibit liver glycosylation and improve peripheral glucose utilization, mainly through the regulation of mitochondrial function. Metformin is widely used to treat diabetes because of its excellent safety and efficacy. Recent studies have shown that metformin has potential antitumor activity, especially in regulating inflammation, metabolism, and cell cycle arrest, and can be used to enhance the antitumor effect of chemotherapy through these effects [3]. Compared with those in normal tissue cells, mitochondrial defects in cancer cells lead to the utilization of glucose mainly through anaerobic glycolysis rather than adenosine triphosphate (ATP) production by glucose through oxidative phosphorylation (OXPHOS) in mitochondria, as in normal cells [4]. Although ATP production in cancer cells is much lower than normal cells, substance utilization can be optimized due to metabolic reprogramming to adapt to this metabolic change [5, 6]. Leukemic cells, like cancer cells, need active mitochondria to survive [7•]. Therefore, it is possible to kill leukemic cells by inhibiting the electron transport chain or mitochondrial translation or by targeting mitochondrial respiratory function. Furthermore, metformin can also affect tumor immunity to achieve antitumor effects [8••]. A recent meta-analysis showed that the long-term use of metformin was associated with a reduction in the incidence of multiple solid tumors [9]. However, studies have shown that diabetic patients who continue to take metformin may have an increased risk of leukemia [10]. This potential cancer-promoting mechanism suggests that the use of metformin may be a double-edged sword for the occurrence of leukemia and is not limited by the known hypoglycemic and antitumor effects, which various from diseases to disease. Considering that the role of metformin in leukemia has not been elucidated, this article further explored the specific mechanism of metformin in the treatment of leukemia to provide a new treatment strategy for this disease.

## Mechanisms by which metformin exerts anticancer effects

AMPK is a widely reported downstream effector of metformin [11]. Metformin inhibits oxidative respiration by acting on complex I of the mitochondrial respiratory chain, thus inhibiting the synthesis of ATP, increasing the adenosine diphosphate (ADP)/ATP ratio and adenosine monophosphate (AMP)/ATP ratio, and promoting the activation of AMPK [12]. In addition, studies have shown that metformin-induced glucose starvation can also lead to the activation of AMPK through the lysosomal v-ATPase-Ragulator complex [13]. AMPK is a key signal integration factor crucial for the control of mitochondrial health and metabolism and is also closely related to cell senescence and cell fate. Studies have shown that metformin-induced AMPK activation can further participate in the regulation of tumor cell growth and apoptosis through a variety of pathways [14, 15]. The activation of AMPK can inhibit the activity of mammalian target of rapamycin (mTOR). mTOR is the active center component of mTOR complex 1 (mTORC1) and mTOR complex 2 (mTORC2), which can co-stimulate cell growth [16, 17]. mTORC1 controls the synthesis of proteins, lipids, and nucleic acids, and mTORC2 can phosphorylate protein kinase B (AKT) to promote cell proliferation [18]. AMPK can also promote the phosphorylation of mTORC2 to block its nuclear translocation and binding to phosphate CRE-binding proteins, thus interfering with the transcription of key gluconeogenesis molecules, such as peroxisome proliferator-activated receptor-γ coactivator-1α (PGC-1α), glucose-6-phosphatase (G6P), and phosphoenolpyruvate carboxykinase (PEPCK) [19]. Metformin blocks the activation of mTORC1 and blocks the initiation of translation, especially the translation of c-myc, cyclin D1, and B-cell lymphoma-extra large (Bcl-xl), which are essential for cancer proliferation [20]. Evidence showed that the RUNX1/signal transducer and activator of transcription 3 (STAT3) also involved in the function of metformin via AMPK pathway [21]. Metformin-induced AMPK activation is closely involved in the regulation of tumor cell fate. For example, a study by Zheng et al. showed that metformin can lead to the overexpression of BAX and tumor cell death through the phosphorylation of AMPK/SIRT1 and the downstream nuclear factor-kappa B (NF-kB) p65 subunit [22]. Metformin can also promote ferroptosis in breast cancer cells by upregulating miR-324-3p [23].

AMPK is a key regulator of cell metabolism and a regulatory molecule of a series of important metabolic enzymes, such as acetyl-CoA carboxylase and HMG-CoA reductase. It can directly regulate the activity of enzymes through phosphorylation. Moreover, the expression of metabolic enzymes at the transcription level can be affected by the phosphorylation of transcription factors and their coactivators, such as acetyltransferase p300. AMPK can participate in the regulation of cell cycle progression through p53 activation and cell metabolism checkpoints and thus participate in the regulation of tumor growth [24, 25]. In addition, AMPK activation can also inhibit ATP formation through fatty acid oxidation (FAO) and stimulate glycolysis through phosphorylation-induced phosphofructokinase-2 (PFK2) [26]. Therefore, interference with glycolysis shed a light on influencing tumor growth.

The anticancer effect of metformin may also be mediated through an AMPK-independent mechanism. It has been reported that metformin can inhibit cell DNA damage by preventing the production of reactive oxygen species (ROS) [27]. In the absence of AMPK, metformin can also affect AKT/mTOR signaling by directly inhibiting mTORC1 signaling [28]. In addition, metformin has been shown to inhibit cyclin D1, an important regulator of the cell cycle [29]. It has been reported that this inhibition is related to the p53-dependent upregulation of Redd1, an effect of the DNA damage response. Redd1 is a negative regulator of mTOR and a new molecular target of metformin [30]. Next, metformin was found to upregulate apoptosis and autophagy through the bcl-2 pathway, thereby inhibiting tumor growth. This effect is mediated by the inactivation of the STAT3/bcl-2 pathway [31]. After the knockout of AMPK, this pathway changed only slightly, indicating that the contribution of AMPK is quite small [31].

The anticancer effect of metformin can also inhibit tumor growth through dietary control, such as reducing blood glucose and insulin resistance and further reducing the levels of insulin and insulin-like growth factor-1 (IGF-1), thus inhibiting the growth of cancer cells [32]. Metformin can weaken the function of hexokinase, which catalyzes the production of G6P, thus inhibiting the utilization of glucose by tumor cells [33]. It has also been reported that metformin reduces glucose uptake in cancer patients. This changes the energy source and energy metabolism of tissues and cells, resulting in improved mitochondrial function [34].

**Fig. 1:**
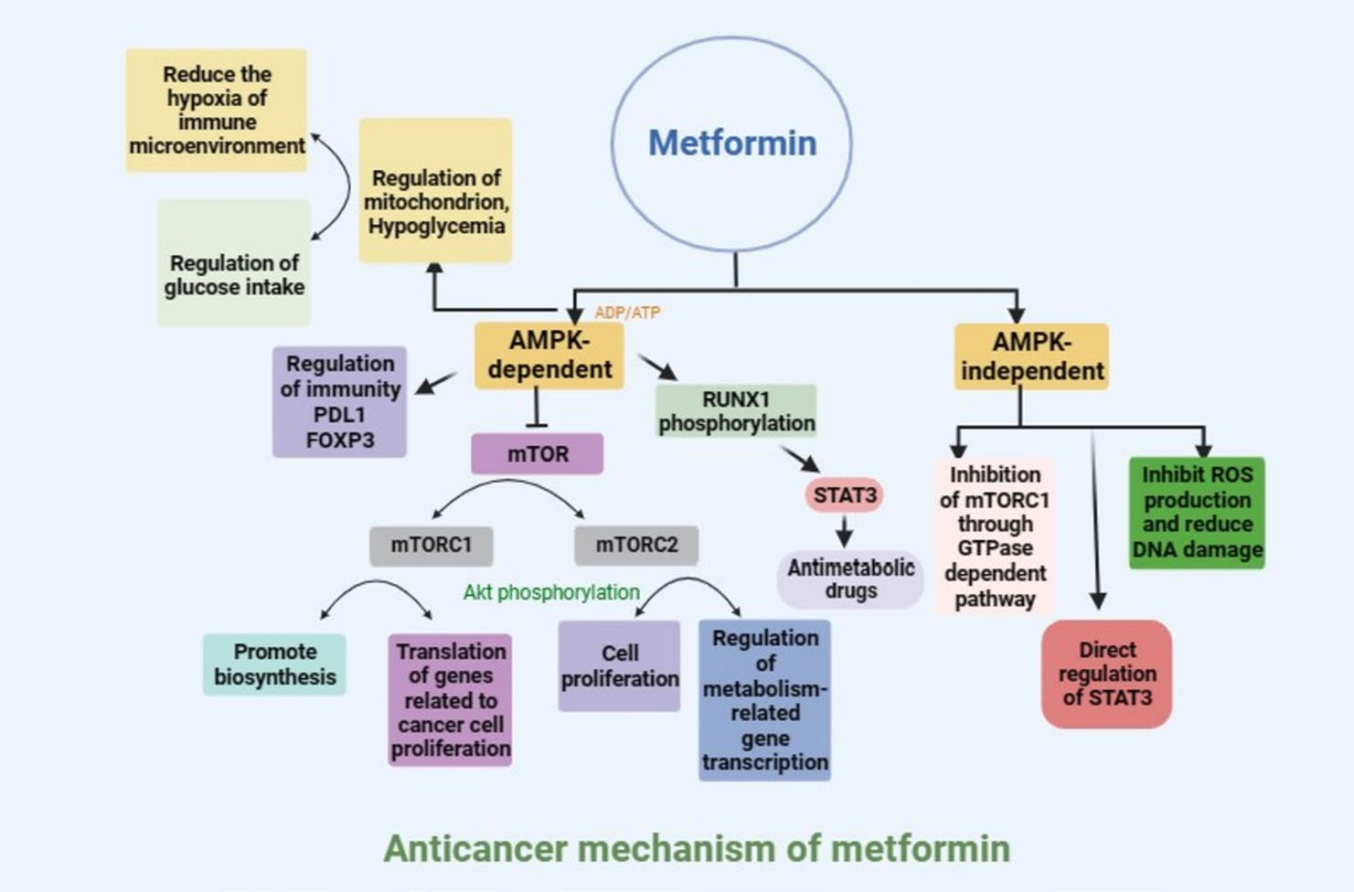
Anticancer mechanism of metformin.

Metformin can also exert its anticancer effect by affecting the immune microenvironment and stimulating the immune response to cancer cells. Metformin potentially ameliorates hypoxia in the tumor microenvironment through its potential effect on mitochondrial function and downstream oxygen production, and it has been reported that metformin can cause the degradation of oxygen-dependent hypoxia inducible factor 1α (HIF-1α) in hepatocellular carcinoma cells, thus improving the phenotype of the inhibitory tumor microenvironment caused by hypoxia [35]. A decrease in HIF-1α levels can also lead to a decrease in CD39 + and CD73 + immunosuppressive myeloid cells in the tumor microenvironment [36]. Metformin can improve immunotherapy resistance by changing the oxygen consumption of small cell lung cancer cells [37]. In addition, metformin may affect the immune response of tumor cells by directly reducing PD1/PD-L1 expression in tumor cells. For example, Cha et al. showed that metformin-induced AMPK activation can increase the degree of phosphorylation of PD-L1 S195 and increase the degradation of PD-L1 in the endoplasmic reticulum, thus reducing the expression level of PD-L1 on the tumor cell surface [38]. AMPK activation can also promote the phosphorylation of the mTOR and S6 proteins and reduce the expression of FOXP3, thus reducing the immunosuppressive function of Treg cells (Fig. 1) [39].

## Research progress on metformin in different types of leukemia

### Acute lymphocytic leukemia (ALL)

ALL is a neoplastic disease characterized by the abnormal malignant proliferation of B or T cells in the bone marrow (BM). Some studies have shown that *PTEN* deficiency in tumor suppressor genes leads to the constitutive activation of the PI3K/Akt/mTOR pathway in T-cell acute lymphoblastic leukemia (T-ALL) [40] and is related to the poor prognosis of patients with T-ALL [41]. Metformin significantly reduces the levels of c-myc and Bcl-xl by stimulating AMPK to inhibit mTOR, thus inhibiting the metabolism and proliferation of cancer cells [42]. In addition, metformin induces the compensatory antiapoptotic activation of Akt and PIM-2, which is reversed by the corresponding inhibitors, which induce cell death when combined with metformin. However, metformin does not affect the survival of normal T lymphocytes, suggesting that metformin can be used to treat ALL [43, 44].

Metformin can also enhance the sensitivity to chemotherapeutic drugs by promoting apoptosis and inhibiting the cell cycle. Clinical data show that the recurrence rate of ALL in a metformin combined with chemotherapy group was lower than that in a chemotherapy group and that this treatment improved the survival rate of patients [45]. Metformin has been shown to enhance the antitumor effect of anthracyclines and reduce growth and survival of ALL cells [46]. In addition to the antitumor effects of synergistic chemotherapeutic drugs, the combination of metformin with chemotherapeutic drugs can help reduce the clinical dose of chemotherapeutic drugs, thus reducing cardiotoxicity and other adverse reactions to anthracyclines [47]. In addition, a clinical study of metformin combined with the chemotherapy drugs vincristine, dexamethasone, doxorubicin and l-asparaginase in the treatment of recurrent childhood ALL (NCT01324180) revealed good clinical effects. The synergistic mechanism may involve metformin inhibiting the unfolded protein response (UPR) and upregulating endoplasmic reticulum stress by activating AMPK, thus mediating the apoptosis of ALL cells [48]. In addition, metformin can enhance the apoptosis of leukemic cells induced by the Bcl-2 inhibitor ABT-737 by inducing mitochondrial membrane depolarization [49].

In vitro cell experiments showed that metformin not only inhibited the malignant proliferation of ALL cells but also reversed drug resistance. Metformin can induce cell cycle arrest and apoptosis in drug-resistant ALL cells [50]. Further studies revealed that high ATP-binding cassette subfamily B member 1 (ABCB1) expression in cancer cells was positively correlated with drug resistance in ALL. The survival rate of patients with high ABCB1 expression is lower than that of patients with low ABCB1 expression, and this difference in survival rate affects patient prognosis. Metformin can increase the sensitivity of ALL patients with high ABCB1 expression to chemotherapeutic drugs, reduce the recurrence rate, and increase the survival rate [51, 52]. Leukemia stem cells (LSCs) are rare cells of leukemic origin and are the cause of leukemia recurrence because they are inherently resistant to chemotherapy [53]. Studies have shown that metformin and chemotherapeutic drugs can achieve antitumor effects by inhibiting the proliferation of cancer stem cells (CSCs), which were also demonstrated in and that ALL model as well [54, 55].

### Chronic lymphocytic leukemia (CLL)

CLL is caused by the clonal accumulation of resting and antiapoptotic malignant B lymphocytes. The tumor cells are monoclonal B lymphocytes that are similar to normal mature small lymphocytes and accumulate in the blood, BM, and lymphoid tissue. In fact, most CLL tumor cells are arrested in the G0/G1 cell cycle stage [56]. Bruno et al. [57] found that metformin induced the death of quiescent CLL cells and inhibited the entry of CLL cells into the cell cycle but had no effect on normal lymphocytes. Metformin induces AMPK phosphorylation, reduces glucose metabolism, and inhibits cell cycle progression in CLL cells [58].

Analyses of glucose dependence in CLL patients showed that different patients had different sensitivities to glucose deprivation. Further evaluation of the metabolic dependence of CLL cells resistant to glucose deprivation showed that glucose transporter type 4 (GLUT4) was upregulated [59]. CLL cells were treated with ritonavir, a human immunodeficiency virus (HIV) protease inhibitor that can inhibit GLUT4, and the toxicity was similar to that caused by glucose deprivation. CLL cells resistant to ritonavir exhibited enhanced drug sensitivity when combined with metformin, potentially by targeting compensatory mitochondrial complex 1 activity [59]. Similarly, metformin has a synergistic effect with the glycolysis inhibitor sodium dichloroacetate (DCA), which can inhibit the expression of the antiapoptotic protein MCI-1, thus promoting CLL cell death [60]. Metformin combined with thymoquinone (TQ) could increase the level of cleaved poly (ADP-ribose) polymerase (PARP) in primary CLL cells, decrease the level of proliferation regulatory proteins, and inhibit the Akt and NF-kB signaling pathways, suggesting that metformin can be used as a therapeutic strategy for CLL [61]. However, the effect and mechanism of action of metformin in CLL are not perfect, and many basic and clinical studies are still needed to determine its therapeutic effect on CLL.

### Acute myeloid leukemia (AML)

AML is a group of heterogeneous malignant diseases characterized by the unrestricted proliferation of BM primitive hematopoietic stem cells (HSCs) that are blocked in the early stage of differentiation. The accumulation of immature undifferentiated cells in the BM and peripheral blood replaces normal terminally differentiated blood cells, leading to progressive anemia, thrombocytopenia, and neutropenia. AML is the most common type of acute leukemia in adults; among those with AML, the majority are males, and the median age is approximately 70 years. The French-American-British (FAB) classification is still in use. The FAB classification is based mainly on the morphological characteristics of leukemic cells [62]. Moreover, there has been no significant change in the clinical treatment of AML in the past 40 years and includes 7–10 days of treatment with cytarabine combined with 3 days of anthracycline treatment as remission induction therapy. In patients younger than 60 years, this treatment yields a complete remission (CR) rate of approximately 60–90%. In addition, several courses of high-dose cytarabine or allogeneic hematopoietic stem cell transplantation (allo-HSCT) can be used as consolidation therapy. Among elderly patients for whom intensive therapy is not suitable, this treatment, including low-dose cytarabine or demethylation therapy and supportive therapy, does not yield good clinical results [63]. At present, new therapeutic drugs for AML, including monoclonal antibodies, small inhibitors, and epigenetic regulators, are in early clinical trials. Currently, the most successful drug therapy for acute lymphoblastic leukemia (APL) is still induced differentiation therapy. APL is a special subtype of AML that is characterized by the translocation of chromosomes 15 and 17. APL is a fatal disease with a high incidence of early hemorrhagic death. It has been successfully treated with all-trans retinoic acid (ATRA) and chemotherapy. The induced CR rate is 90%, and the cure rate is approximately 80%. In the later stage, arsenic trioxide can be introduced for the treatment of refractory or recurrent APL, and better clinical results have been obtained [64]. Huai et al. reported that metformin inhibits the proliferation and differentiation of the APL cell line NB4 by inhibiting extracellular regulated protein kinase (ERK), live kinase B1 (LKB1)/AMPK, and other signaling pathways to activate the apoptosis-related protease caspase-3, subsequently promoting apoptosis and reducing tumor cell adhesion [65].

In a recent retrospective study, the use of metformin did not significantly improve the overall disease-free survival of patients with AML [66]. Compared with normal cells, leukemic cells exhibit high levels of AKT phosphorylation, glucose consumption, and glycolysis, which may reduce the metformin-induced Pasteur effect and induce resistance to metformin-induced apoptosis. Therefore, treatment with deoxyglucose or AKT inhibitors can increase the sensitivity of leukemic cells to metformin. Therefore, when cultured in low-glucose medium or when glycolysis is downregulated by 2-DG or AKT inhibitors, AML cells may be more sensitive to metformin [67]. Liu et al. showed that metformin can inhibit OXPHOS. AML cells with the MLL/AF9 genotype are highly dependent on oxidative phosphorylation and can be targeted by metformin. Metformin significantly inhibits the proliferation of MLL/AF9 AML cells by inhibiting mitochondrial respiration [68]. In AML, metformin can block the expression of proto-oncogenes by inhibiting mTOR, inhibiting the proliferation of G0/G1 or S-G2/M cells, interfering with cancer cell proliferation and colony formation activity, and inducing apoptosis but not affecting the proliferation and differentiation of normal HSCs [20]. Numerous in vitro experiments have confirmed that metformin, an AMPK agonist, can inhibit the growth of the AML cell lines OCI/AML2, OCI/AML3, and THP-1 in vitro; is a potential drug for adjuvant therapy for leukemia; and inhibits the growth and survival of chronic myeloid leukemia (CML) cells expressing BCR-ABL mutations in vitro [69, 70]. The inhibitory effect of metformin has also been observed in the phorbol-12-myristate-13-acetate (PMA)-mediated differentiation of the acute monocytic leukemia cell line THP-1 [71]. The role of metformin in AML may depend on the basic activity of mTOR and ERK, which are downstream targets of AMPK [72]. In addition, metformin-mediated apoptosis and inhibition of proliferation in AML cells were preserved when AMPK expression was downregulated by small interfering RNA (si-RNA), indicating that metformin may have an AMPK-independent regulatory effect on AML [67, 73]. Increasing evidence shows that the different effects of metformin on hematopoietic and immune cell differentiation and its subsequent immunomodulatory effect may be another mechanism by which metformin exerts antitumor activity [74].

In addition to the direct inhibitory effect of metformin on AML, metformin can also be used as an adjuvant to enhance the therapeutic efficacy of treatment for AML. First, some studies have shown that metformin can inhibit the transformation of stromal cells into AML cells by blocking mitochondria, a process that can significantly enhance the sensitivity of AML cells to small molecule chemical drugs [75–77]. Second, metformin can also inhibit mTORC1 and increase the sensitivity of AML to cytarabine through its classical regulatory pathway [78]. The antileukemic effect of the Flt3 inhibitor sorafenib was investigated in FLT3-ITD-positive AML cells, which are associated with poor prognosis. Metformin enhances the antileukemia activity of sorafenib by downregulating the expression of related genes in the mTOR pathway [79]. In addition, in the study of AML, it has been found that metformin combined with the nonsteroidal anti-inflammatory drugs diclofenac and diflunisal can induce the apoptosis of AML cells [80].

Recently, as the only Bcl-2 selective inhibitor approved for sale worldwide, venetoclax was approved by the Food and Drug Administration (FDA) for use in newly diagnosed AML patients, especially those who are not suitable for routine chemotherapy. Although venetoclax has been shown to have ideal therapeutic efficacy in clinical practice, the acquisition of drug resistance induced by the activation of the apoptosis pathway by ABT-199 is still an important clinical problem. For this reason, Zhou et al. confirmed in vitro and in vivo that compared with metformin or ABT-199 alone, the combination of these two drugs has synergistic effects on promoting apoptosis, resulting in greater antileukemia effects [58]. In addition, the combined use of metformin and ABT-199 significantly decreased the expression of the apoptosis-related protein myeloid cell leukemia-1 (Mcl-1) induced by ABT-199 alone and inhibited the expression of another antiapoptotic protein, BCL-xl, to some extent [58].

### Chronic myeloid leukemia (CML)

CML is characterized by the production of many immature white blood cells, which accumulate in the BM and inhibit normal hematopoiesis in the BM. It often occurs in adults between the ages of 40 and 60 years. The cause is usually the rearrangement of two designated chromosomes (chromosomes 9 and 22) to form the so-called Philadelphia chromosome. The Philadelphia chromosome encodes an abnormal enzyme, tyrosine kinase, which leads to an increase in the number of white blood cells produced in abnormal growth patterns in patients with CML [81]. Tyrosine kinase inhibitors (TKIs) are first-line treatments for CML. A complete cytogenetic response (CCyR) has been shown to be associated with increased overall survival, with only 66% of patients reaching CCyR after 1 year of TKI treatment [81]. In preclinical studies, metformin has been shown to inhibit the viability of imatinib-resistant CML cells (K562R) and BCR-ABL-mutant CML cells, induce apoptosis, and downregulate the mTORC1 signaling pathway [82]. In a clinical study, metformin and TKIs can increase the proportion of patients with CML to achieve CCyR and shorten the time to reach a major molecular response (MMR) or complete molecular response (CMR) [83]. Nilotinib is used to treat patients with imatinib-sensitive or drug-resistant CML. However, in recent years, there have been cases of nilotinib resistance. Researchers have found that the nilotinib-resistant CML cell lines K562 and KU812 are produced by exposing cells to gradually increasing doses of nilotinib. Several studies have shown the driving force of resistance to nilotinib and the effect of metformin on the driving force. Metformin can enhance the apoptosis of CML cells induced by nilotinib and restore the sensitivity of drug-resistant cells to nilotinib by reducing Jun N-terminal kinase (JNK) phosphorylation and inhibiting Bcl-xl expression. The results of the above studies suggest that the combination of metformin and nilotinib may have enhanced the efficacy of leukemia treatments and overcome resistance to nilotinib [84]. Metformin can also inhibit the growth and promote the apoptosis of the human CML cell line K562 by inhibiting glycolysis and the PI3K/Akt/mTOR pathway. Metformin may be a promising adjuvant for the treatment of CML (Table 1) [85].

**Table 1:** Research progress on the use of metformin in different types of leukemia

Type of leukemia	Key findings	Combination drug	Reference
ALL	Induces apoptosis through AMPK-dependent inhibition of UPR signaling	-	[44]
	Inhibits mTOR signaling to enhance the antitumor effect	Anthracycline	[46]
	Inhibits the UPR and upregulates endoplasmic reticulum stress by activating AMPK	Vincristine, dexamethasone, PEG-asparaginase, doxorubicin	[48]
	Induces mitochondrial membrane depolarization to enhance apoptosis	ABT-737	[49]
	Inhibits cell proliferation by arresting cells in the G2/M and S phases of the cell cycle	-	[50]
CLL	Induces AMPK phosphorylation and reduces glucose metabolism	Fludarabine, ABT-737	[57]
	Downregulates the expression of Mcl-1 and Bcl-xl by inhibiting protein synthesis	ABT-199 (venetoclax)	[58]
	Potentially targets compensatory mitochondrial complex 1 activity to sensitize CLL cells resistant to ritonavir	Ritonavir	[59]
	Induces synergistic apoptotic cell death coupled substantial Mcl-1 protein downregulation	DCA	[60]
	Increases the level of cleaved PARP and inhibits the AKT and NF-kB signaling pathways	TQ	[61]
AML	Inhibits ERK, LKB1/AMPK and other signaling pathways to activate caspase-3	ATRA	[65]
	Decreases electron transport chain complex I activity, oxygen consumption, and mitochondrial ATP synthesis while stimulating glycolysis for ATP and metabolic effects	Deoxyglucose, AKT inhibitor	[67]
	Represses cell proliferation by inhibiting mitochondrial respiration	-	[68]
	Blocks the expression of proto-oncogenes by inhibiting mTOR and then inhibits the proliferation of cells in the G0/G1 or S-G2/M phase	-	[20]
	Downregulates the expression of TYRO3 and phosphorylation of MERTK, partly due to the inhibition of TAM kinases	TP-0903 (a small molecule AXL inhibitor)	[70]
	Inhibits THP-1 macrophage-derived foam cell formation induced by LPS, reduces intracellular lipid accumulation, and downregulates the expression of ADRP	PMA	[71]
	Inhibits the mitochondrial transfer and OXPHOS activity	Ara-C	[75]
	Inhibits the mTORC1/P70S6K pathway and increases the sensitivity of AML to Ara-C	Ara-C	[78]
	Increases LC3 levels and reduces the expression of proteins in the mTOR/p70S6K/4EBP1 pathway, without appreciably altering the cell cycle	Sorafenib	[79]
CML	Suppresses survival, induces apoptosis, and downregulates the activation of the mTORC1 pathway	Imatinib	[82]
	Reduces Bcl-xl expression, decreases phosphorylated JNK levels, and restores nilotinib sensitivity	Nilotinib	[84]
	Inhibits the growth and proliferation of K562 cells and promotes the apoptosis of K562 cells by inhibiting glycolysis energy metabolism	-	[85]

*ALL* acute lymphocytic leukemia, *AMPK* adenosine 5′-monophosphate (AMP)-activated protein kinase, *UPR* unfolded protein response, *mTOR* mammalian target of rapamycin, *CLL* chronic lymphocytic leukemia, *Mcl-1* myeloid cell leukemia-1, *Bcl-xl* B-cell lymphoma-extra large, *DCA* dichloroacetate, *PARP* poly(ADP-ribose) polymerase, *AKT* protein kinase B, *NF-kB* nuclear factor-kappa B, *TQ* thymoquinone, *ERK* extracellular regulated protein kinases, *LKB1* live kinase B1, *AML* acute myeloid leukemia, *ATRA* all-trans retinoic acid, *ATP* adenosine triphosphate, *TYRO3* protein tyrosine kinase gene, *MERTK* tyrosine-protein kinase mer, *TAM* tumor-associated macrophage, *LPS* lipopolysaccharide, *PMA* phorbol-12-myristate-13-acetate, *ADRP* adipose differentiation-related protein, *OXPHOS* oxidative phosphorylation, *mTORC1* mTOR complex 1, *P70S6K* phosphoprotein 70 ribosomal protein S6 kinase, *Ara-C* cytarabine, *LC3* microtubule-associated protein 1 light chain 3, *4EBP1* eukaryotic initiation factor 4E-binding protein 1, *CML* chronic myeloid leukemia, *JNK* Jun N-terminal kinase

## Clinical trials

Although metformin may theoretically be used as a potential adjuvant for a variety of leukemias and many preclinical in vitro or in vivo trials provide supporting data, there is still a lack of high-quality evidence from clinical trials. Most of the existing clinical studies have focused on the application of metformin in ALL patients. In a randomized controlled study (NCT00500240) reported in 2012, Khanh et al. studied the hypoglycemic effect of metformin in patients with diabetic ALL and reported that metformin not only had a hypoglycemic effect but was also associated with improved PFS; moreover, multivariate analysis revealed that blood glucose level was an independent risk factor for OS in patients with ALL [86]. In 2014, a randomized controlled trial in Spain showed that metformin can lead to a 56% reduction in the risk of relapse according to Cox regression analysis [87]. In 2018, another randomized controlled study (NCT03118128) by Christian et al. showed that for ALL patients with high ABCB1 expression, metformin combined with the LALHGM07 chemotherapy regimen improved survival and reduced the risk of treatment failure (OR = 0.07, 95% CI = 0.0037–1.53) and early recurrence (OR = 0.05, 95% CI = 0.0028–1.153). It is suggested that metformin can be used as an adjuvant therapy for specific patient subgroups [52]. In addition, two related clinical studies are underway, one focusing on the potential therapeutic value of metformin for adolescent ALL (NCT05326984) and the expression of biomarkers and the other on the effect of metformin on the risk of leukemia in patients with clonal cytopenia of undetermined significance (CCUS) or lower-risk myelodysplastic neoplasms (LR-MDS). We also look forward to obtaining additional evidence-based medicine data to support the clinical application of metformin in patients with leukemia in the future. We also hope that additional studies will focus on patients with types of leukemia other than ALL to provide insight into the clinical application of metformin in leukemia treatment (Table 2).

**Table 2:** Clinical trials

Clinical trial ID	Official title	Status
NCT05326984	Effect of metformin on ABCB1 and AMPK expression in adolescents with newly diagnosed ALL	Recruiting
NCT04741945	Repurposing metformin as a leukemia-preventive drug in CCUS and LR-MDS	Recruiting

*ABCB1* ATP**-**binding cassette subfamily B member 1, *AMPK* adenosine 5′-monophosphate (AMP)-activated protein kinase, *ALL* acute lymphocytic leukemia, *CCUS* clonal cytopenia of undetermined significance, *LR-MDS* lower-risk myelodysplastic neoplasms

## Conclusions and perspectives

Metformin is a safe and inexpensive small molecule antidiabetic drug that has been used for the treatment of diabetes for several decades. In recent years, metformin has aroused widespread interest for its antitumor, immunoregulatory, antiaging, and other effects. To date, numerous medical studies have suggested that metformin is beneficial for patients with leukemia, but most of these studies are limited to preclinical studies, and the results of a small number of clinical trials are also different. In our previous in vitro study, we found that venetoclax and metformin alone inhibited the proliferation of AML cells, promoted apoptosis, and reduced the mitochondrial membrane potential. The effects of inhibiting proliferation and promoting apoptosis were time and concentration dependent. In animal experiments in the same study, metformin combined with venetoclax reduced the leukemia load and prolonged the survival of AML xenograft mice. Therefore, studying the pharmacological effects of metformin and the efficacy of metformin in treating hematopoietic diseases may also be helpful for understanding the pathophysiological process of the occurrence and development of hematological diseases. Therefore, determining whether metformin can be used as a therapeutic drug for leukemia and how to correctly evaluate its clinical efficacy still require a large amount of new data. In the future, scholars should conduct more in-depth research on the role of metformin in combination with other anticancer drugs.

## Data Availability

No datasets were generated or analyzed during the current study.
